# Women's Empowerment and Contraceptive Use: The Role of Independent versus Couples' Decision-Making, from a Lower Middle Income Country Perspective

**DOI:** 10.1371/journal.pone.0104633

**Published:** 2014-08-13

**Authors:** Waqas Hameed, Syed Khurram Azmat, Moazzam Ali, Muhammad Ishaque Sheikh, Ghazunfer Abbas, Marleen Temmerman, Bilal Iqbal Avan

**Affiliations:** 1 Research, Monitoring and Evaluation Department, Marie Stopes Society, Karachi, Sindh, Pakistan; 2 Department of Uro-gynecology, University of Ghent, Ghent, East Flanders, Belgium; 3 Department of Reproductive Health and Research, World Health Organization, Geneva, Switzerland; 4 Faculty of Infectious and Tropical Diseases, London School of Hygiene and Tropical Medicine, London, United Kingdom; Karolinska Institutet, Sweden

## Abstract

**Introduction:**

There is little available evidence of associations between the various dimensions of women's empowerment and contraceptive use having been examined - and of how these associations are mediated by women's socio-economic and demographic statuses. We assessed these phenomena in Pakistan using a structured-framework approach.

**Methods:**

We analyzed data on 2,133 women who were either using any form of contraceptive or living with unmet need for contraception. The survey was conducted during May - June 2012, with married women of reproductive age (15–49 years) in three districts of Punjab. The dimensions of empowerment were categorized broadly into: economic decision-making, household decision-making, and women's mobility. Two measures were created for each dimension, and for the overall empowerment: women's independent decisions, and those taken jointly by couples. Contraceptive use was categorized as either female-only or couple methods on the basis of whether a method requires the awareness of, or some support and cooperation from, the husband. Multinomial regression was used, by means of Odds Ratios (OR), to assess associations between empowerment dimensions and female-only and couple contraceptive methods.

**Results:**

Overall, women tend to get higher decision-making power with increased age, higher literacy, a greater number of children, or being in a household that has superior socio-economic status. The measures for couples' decision-making for overall empowerment and for each dimension of it showed positive associations with couple methods as well as with female-only methods. The only exception was the measure of economic empowerment, which was associated only with the couple method.

**Conclusion:**

Couples' joint decision-making is a stronger determinant of the use of contraceptive methods than women-only decision-making. This is the case over and above the contribution of women's socio-demographic and economic statuses. Effort needs to be made to educate women and their husbands equally, with particular focus on highly effective contraceptive methods.

## Introduction

Worldwide, 292,982 women died in 2013 due to pregnancy-related complications; and more than 50% of all maternal deaths were in just six developing countries, including Pakistan [1], [2]. These deaths are projected to be 1.8 times higher in women without contraceptive use [3]. Among many interventions, contraceptive use to prevent unwanted pregnancies is one of the most cost-effective ways of reducing maternal deaths [4].

The health of women and their children in many societies is adversely affected by women's inferior social status within households. This is mainly because of the culturally and socially determined roles for women that pervade every aspect of their lives [5], [6]. Women in South Asia sacrifice their desire to regulate their fertility because they are nurtured in such a way that their family-group interest supersedes their personal desire [7]. Consequently, women's empowerment is recognized as an imperative element in enabling couples to access reproductive health services - including family planning - for improved mother-and-child health [8]. It is suggested that gender-based control in relationships is associated with sexual- and reproductive-health outcomes [9].

The concept of women's empowerment is complex, as there is considerable variation in its conceptualization. Most definitions link empowerment with the power or freedom used to achieve desired outcomes. For example, Safilos-Rothschild defines “women's status as women's overall position in the society while ‘power’ refers to women's ability to influence and control at the interpersonal level” [10]. Krishna describes women's empowerment as “the process of increasing the capacity of women to make choices and to transform these choices into desired actions and outcomes” [11]. In South Asia, empowerment is referred to as “the process in which women challenge the existing norms and culture to effectively improve their well-being” [12]. Nabeela Kabeer notes “the expansion in women's ability to make strategic life choices in a context where this ability was previously denied to them” as empowerment. According to the Department for International Development (DFID), empowerment is “a process of transforming gender relations through groups or individuals developing awareness of women's subordination and building their capacity to challenge it” [13].

The dynamic and multidimensional nature of women's empowerment, and its existence at various levels, make it challenging for researchers to measure empowerment [14]. Several proxy indicators are used to measure empowerment, such as education and employment status; however, though related to it, these characteristics do not reflect empowerment [15], [16]. Offering one of the most comprehensive frameworks for measuring women's empowerment, Malhotara et al. propose measuring the general development of empowerment at different levels, and in six categories or dimensions: economic, socio-cultural, familial/interpersonal, psychological, legal, and political [15]. There is considerable literature from several countries on the relationship between women's empowerment and the use of contraception [9], [17]–[22]. However, most studies in this area have examined the effect of either only a single-aspect dimension or an overall empowerment on contraceptive use; yet analysis has shown that not all dimensions of women's empowerment correlate equally with contraceptive use [17]. There is insufficient evidence in the literature available to establish a relationship between the empowerment of women and contraceptive use in Pakistan [22], [23]. Moreover, to the best of the authors' knowledge, there is little available evidence of having assessed the mediating effects of women's socio-economic and demographic statuses on the relationship between empowerment and contraceptive use.

**Figure 1:**
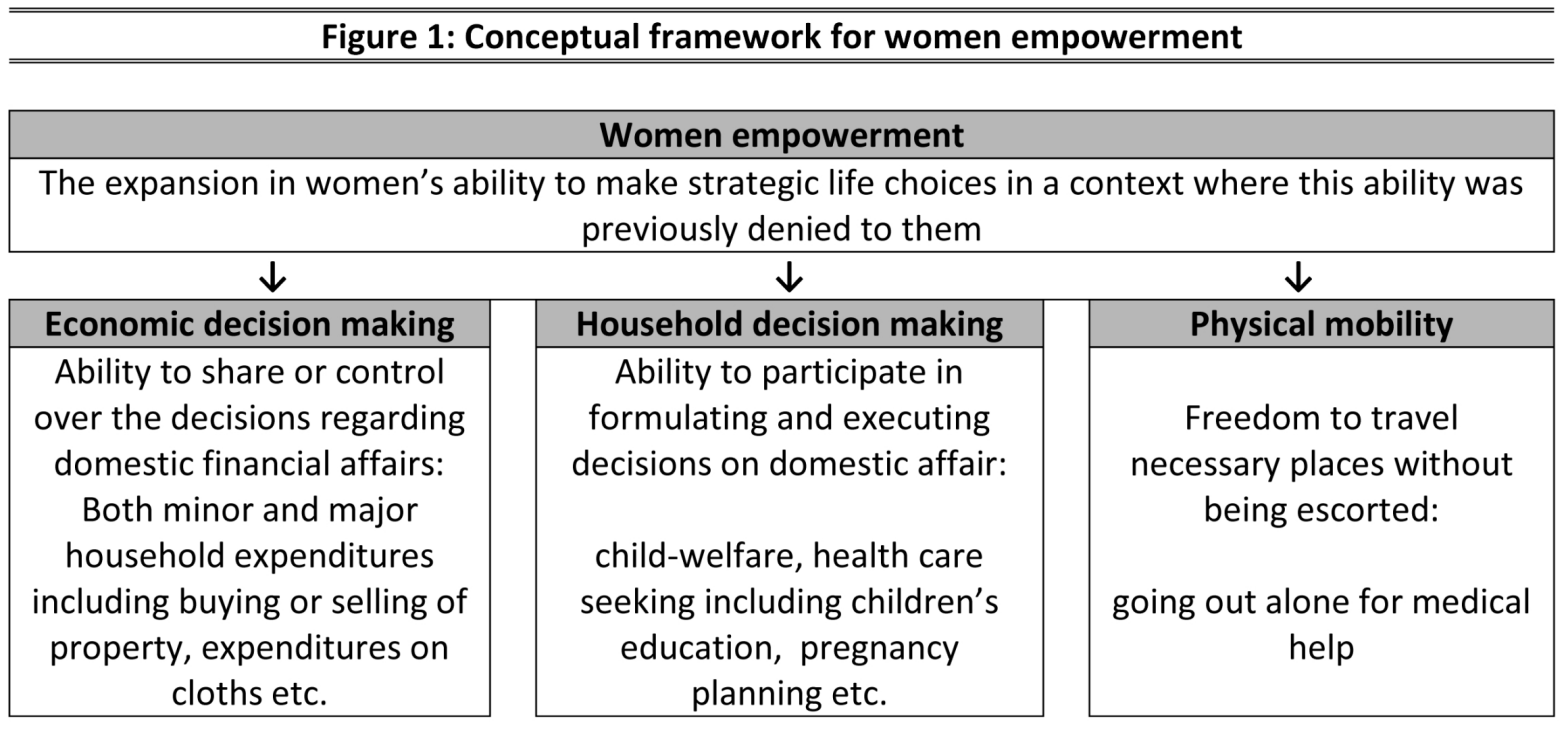
Conceptual framework for women empowerment.

Mindful of the implicit assumption that in most societies, particularly in Asia, men control the women of their social class - especially in their households and families - we chose the operational definition of empowerment proposed by Kabeer [24]. This is because it is most widely accepted, and addresses, in particular, the individual level instead of group- or community-level empowerment. Above all, it effectively covers nearly all the key dimensions in the stated definitions. In this paper, we have aimed to assess the overall effect of women's empowerment and its various dimensions on contraceptive use by adapting the structured framework used by Haque et al. [25] in Bangladesh. We hypothesized that empowered women are more likely to practise contraception, compared with women who are not (or are less) empowered. Specifically, we examined the effect of empowerment on female-only methods, as well as couple methods in which the husband's involvement or support are necessary. The framework we used consisted of three key dimensions: economic decision-making, household decision-making, and women's physical mobility (see figure 1). In addition, we attempted to determine the mediating effects of women's socio-economic and demographic characteristics on the relationship in question.

### Country setting

Pakistan has a population of over 180 million [26] with a sex ratio of 102 males per 100 female [27], 65% of the people live in rural areas [28], 20% of women or couples want to delay or limit their pregnancies; and nearly one in the total fertility rate (TFR) of 3.8 is an unwanted birth, with a median birth-spacing time of 28 months [27]. Current use of modern contraception is around 26%, with the most common methods being either permanent (female sterilization), less effective (condom), and traditional (withdrawal) [27]. In Pakistan, the patriarchal framework of society works at all levels to place women in a more vulnerable position than men [5]. According to the 2012 Gender Gap Report, Pakistan scores almost lowest among the countries assessed on gender disparities, ranking 134 out of 135 countries [29]. In order, also, to improve key maternal- and reproductive-health indicators, addressing the issue of women's empowerment is recommended [30].

## Methods

### Study setting and sample

This paper reports on the baseline-survey data collected during May - June 2012, as part of a larger study underway in three districts (Chakwal, Mianwali and Bhakkar) of Punjab, Pakistan; each district has a total population of approximately 1.3 million [31]. The study aims to assess and evaluate the effectiveness of demand-side family-planning approaches in promoting the healthy timing and spacing of pregnancies [32].

A total of 3,998 interviews were carried out in the catchment areas of 41 primary healthcare facilities (24 located in rural and 17 in urban areas) for the provision of family-planning services in the aforementioned districts of Punjab. The healthcare facilities were selected based on set criteria. Prior to data collection, all households located within a 4–7-kilometres radius around each selected healthcare facility were independently allotted a unique identifier. The household numbering was used for population estimation around each facility. Then, employing the probability proportional to size (PPS) technique, the sample was proportionally distributed across the selected health facilities. Finally, using Epi-info [33], households were randomly selected from the lists, and within each selected household the youngest eligible woman was invited to participate in the survey. Local area enumerators were hired, and trained on the questionnaire. Face-to-face interviews were conducted at participants' homes in privacy. Data were double-entered using Visual FoxPro Version 6.0 (Microsoft Corporation, Redmond, WA, USA). In order to align with the definition of female-only and couple methods, we excluded male-sterilization cases from the analysis (n = 7). Moreover, from a group of non-users we further excluded cases (n = 1858) by restricting our sample to those women who had an unmet need of contraception (i.e. those who wanted to limit or space pregnancies, and were sexually active but not using any form of contraceptive method). Consequently, analyses were performed on a total of 2,133 cases (see Table S1 regarding contraceptive use and unmet need for contraception based on original sample). The non-response rate documented during the survey was 3%. Among the main reasons for non-response were: women's unwillingness, as they were busy with household chores; and family members not allowing the sharing of information for reasons to do with security, a mistrust of authorities and the poor law-and-order situation locally.

### Eligibility criteria

The survey participants were currently married women of reproductive age (15–49), residents of the catchment area, neither mentally nor physically handicapped, and not sick.

### Ethical consideration

The study protocol was approved by the National Bioethics Committee (NBC) Pakistan. Ref: No. 4-87/12/NBC-92/RDC/3548. All survey participants provided a written informed consent [32].

## Measures

### Female-only and couple contraceptive methods

We categorized contraceptive methods into female-only and couple methods. The fundamental difference between the two is that for couple methods – which included male condom, withdrawal, and periodic abstinence (periodic abstinence included standard days methods, cervical mucus method, and calendar method) – women require at least the awareness of, or a certain degree of support, involvement or cooperation from, the husband. Female-only methods included the pill, injectable, IUCD, and implant.

### Outcome variables

The outcome variable was current contraceptive use, which we classified into three categories: 0 =  non-users; 1 =  female-only methods (pill, injection, intra-uterine device, implant, and female sterilization); 2 =  couple methods (condom, withdrawal and periodic abstinence).

### Construction of women's empowerment indices in decision-making

We included thirteen questions regarding household decision-making which were proposed in the framework as well as used in several other research studies [22], [23], [34]–[40]. Based on the conceptual framework (figure 1), these items were classified into three broad dimensions. Economic Decision-making: (1) buying or selling of property, (2) small household expenditures (e.g. toothpaste, soap, crockery etc.), (3) major household expenditures (e.g. TV, refrigerator, furniture etc.), (4) expenditures on woman's clothes, cosmetics, jewelry etc., (5) purchase of medicines, and (6) children's clothes; Household Decision-making: (1) where to go outside for medical care in the event of the woman's illness, (2) where to take the child in the event of illness, (3) children's education, and (4) when to plan pregnancy; and Physical Mobility: (1) visiting relatives, (2) woman's employment outside the home, and (3) woman going out of the house alone for medical help. The responses to all indicators were categorized broadly as ‘woman decides independently’, ‘husband decides independently’, ‘husband and woman decide together’, ‘mother-in-law decides’, ‘woman and mother-in-law decide together’, and ‘other family members decide’. The only exception was that the indicator ‘women's mobility outside home alone for medical help’ was recorded as a dichotomous variable.

We constructed two separate measures for overall women's empowerment, as well as for each of the empowerment dimensions as proposed in the framework above (figure 1). The classification of the two measures was based on *‘women's independent’* and *‘couples’ joint’* decision-making on the aforementioned indicators. For the measure of overall empowerment for independent decision-making, we converted all indicators into binary variables where women were coded as ‘1’ if they decided on the above 13 items *independently*, and ‘0’ otherwise. Because the number of indicators varied from one empowerment dimension to the next - for example, the measurement of economic empowerment was based on six indicators, household empowerment on five, and the dimension of physical mobility on three – the contributions to the overall empowerment composite score were uneven. Therefore, we gave additional weight to the dimensions of ‘household decision-making’ and ‘physical mobility’ to reflect their equal contribution to the composition of the overall empowerment measure. Thereafter, we constructed a composite score by adding the number of decisions. The total score ranged from 0–18, where a high score reflected a higher degree of decision-making power. The same procedure was repeated for the index of couples’ decision-making, where cases were coded as ‘1’ if couples took a *joint* decision, and ‘0’ otherwise. Using a similar strategy, we constructed two measures for each of the three dimensions of women's empowerment. The scores for household decision-making, economic decision-making and physical mobility ranged from 0–6, 0–4 and 0–3, respectively. The 13-item indexes for *independent* and *joint* decision-making were highly consistent, internally-reliable measures for women's empowerment with Kuder Richardson's value of 0.83 and 0.89, respectively.

### Potential confounders

Included in the analysis were a range of independent variables that had the potential to be confounders. These variables mainly depict women's status in the society, and they include: women's and their husbands' age and literacy; the number of children; the age difference between women and their husbands; the age of women at the time of marriage; and socio-economic status in the form of wealth quintiles. We constructed wealth quintiles using principal-component analysis in a manner similar to its original application in demographic and health surveys. The first component score was stored and categorized in quintiles.

### Statistical analyses

Using SPSS 17.0 (SPSS Inc. Released 2008. SPSS Statistics for Windows, Version 17.0. Chicago: SPSS Inc), we carried out the analyses in multiple phases. First, using the mean and proportion, we described the women and the household's characteristics - women's overall empowerment and its different dimensions. Second, we applied bivariate analyses, by means of Pearson's chi-square, to investigate the factors associated with women's overall empowerment. Finally, using the multinomial logistic regression technique, we applied a block-modeling strategy whereby each group of variables is entered into a model separately, resulting in eight different models: (1) a crude model, to examine whether overall empowerment and its dimensions are significantly associated with contraception; (2) a partially-adjusted model in which the wealth quintile was included, to see if the association remained after adjusting for possible confounders; and (3) a final model in which variables related to women's socio-demographic characteristics were added to determine whether the association was explained by women's statuses. The role of women's age and wealth quintile as effect modifier was measured separately by testing the interaction with each empowerment measure. P-value of <0.05 were considered significant. Statistical weights were used to adjust the representation of the population of three districts, and the rural and urban populations. The population adjustment was made using the 2011 projected population of each district.

## Results

### Descriptive analysis

#### Characteristics of the study population

Of the women interviewed, the majority (50.5%) were aged above 35 years, and two fifths (39.6%) had 3–4 living children. Approximately half of the women's husbands were aged above 40 years; and, notably, 84.4% of the women were at least one year younger than their husbands. Nearly three out of five women (59.3%), and a quarter of their husbands (27.2%), were illiterate. Only 29.9% of the women reported to be users of any contraceptive method at the time of interview – including 19.7% female-only and 12.0% couple methods (please see appendix 1 for contraceptive method-mix and unmet need based on original sample). With regard to empowerment, 44.5% of the women reported having the power to take at least one decision independently, and 62% took at least one joint decision with their husbands (table 1). Distributions of women's independent and couples' decision-making were right-skewed (x-axis: number of decisions; y-axis: percentage of women), with more women having low decision-making autonomy (results not shown).

**Table 1:** Characteristics of the sample (n = 2133).

Variables	%	Variables	%
**Women's age**		**Women's literacy**	
≥15–<25 years	7.9	Illiterate	59.3
≥25–<35 years	41.7	Literate	40.7
≥35 years	50.5	**Husband's literacy**	
**Age of women at marriage**		Illiterate	27.2
<20	76.3	Literate	72.8
≥20–<25	19.8	**Number of living children**	
≥25–35	3.9	0	2.6
**Husband's age**		1–2	21.6
<30 years	12.2	3–4	39.6
≥30–<40 years	36.9	5+	36.2
≥40 years	50.9	**Current use of contraception**	
**Age difference**		No	68.3
Women is older	5.5	Female-only methods (pill, injectable, Intra-uterine device, implant, female sterilization)	19.7
Same age (−1 to +1 range)	10.1	Couple methods (condom, withdrawal, periodic abstinence)	12.0
Women is younger	84.4	**Overall Women empowerment**	
		At least one independent decision	44.5
		At least one decision joint decision with husband	62.0

Table 2 elicits that women's empowerment was observed as highest in the economic domain where 32.7% of women reported taking at least one economic decision independently, while the majority (46.3%) of the couples tended to take at least one joint decision with regard to physical mobility. Similarly, 23.5% of the women took at least one decision regarding domestic affairs independently, and 42.8% decided jointly with their husbands.

**Table 2:** Women empowerment in different household decision making (n = 2133).

Dimensions of women empowerment	Independent decision %	Couple decision %	Dimensions of women empowerment	Independent decision %	Couple decision %
**Household decision making**	**23.5**	**42.8**	Visiting relatives	7.9	36.0
When to plan a pregnancy	7.4	15.7	**Economic decision making**	**32.7**	**41.2**
Choosing source of healthcare in case of woman illness	11.6	23.0	Buying or selling of property	2.1	12.2
Choosing source of healthcare for child in the case of illness	12.7	23.6	Major household expenditures (e.g. TV, refrigerator, etc.)	6.0	12.6
Children's education	9.0	26.0	Small household expenditures (e.g. toothpaste, soap, crockery etc.)	15.3	13.2
**Physical mobility**	**21.7**	**46.3**	Purchase of medicines	8.4	19.2
Woman go out alone for medical help (yes/no)	14.4	-	Children's clothes	21.5	27.9
Woman's employment outside the home	1.9	12.5	Expenditures on woman's clothes and jewelry etc.	19.1	28.4

#### Factors related to women's empowerment

The association between the sample characteristics and women's empowerment is shown in table 3. The analysis revealed that women's literacy, the number of living children and the wealth quintile were positively associated with both measures of women's empowerment. The ages of the women and their husbands were also found to be correlated with independent decision-making. Literate husbands were more likely to make joint decisions with women. Overall, women tend to get higher decision-making power with increased age, higher literacy, a higher number of children, or being in a household that has superior socio-economic status.

**Table 3:** Univariable analysis of women's empowerment in decision making and socio-economic and reproductive health characteristics (n = 2133).

Variables	Independent decision %	Couple decision %
**Women's age**		
≥15–<25 years	33.3	58.1
≥25–<35 years	41.7	63.8
≥35 years	48.4	61.1
*P-value*	*<0.001*	*0.265*
**Age of women at marriage**		
<20	44.1	62.4
≥20–<25	44.1	62.3
≥25 +	53.0	51.8
*P-value*	*0.275*	*0.152*
**Husband's age**		
<30 years	35.4	55.8
≥30–<40 years	39.5	62.2
≥40 years	50.2	63.3
*P-value*	*<0.001*	*0.081*
**Age difference**		
Women is older	47.0	60.7
Same (−1 to +1 range)	50.7	65.6
Women in younger	43.6	61.6
*P-value*	*0.117*	*0.502*
**Women's literacy**		
Illiterate	40.1	56.0
Literate	50.8	70.6
*P-value*	*<0.001*	*<0.001*
**Husband's literacy**		
Illiterate	41.2	54.9
Literate	45.7	64.6
*P-value*	*0.064*	*<0.001*
**Number of living children**		
0	38.6	64.9
1–2	40.1	67.2
3–4	48.6	62.4
5+	42.9	58.1
*P-value*	*<0.011*	*0.014*
**Wealth quintile**		
1^st^ (Poorest)	35.3	52.1
2^nd^	33.9	59.9
3^rd^	47.4	65.6
4^th^	40.9	59.8
5^th^ (Least poor)	62.1	70.2
*P-value*	*<0.001*	*<0.001*

#### Effect of women's empowerment on contraception

Table 4 shows the results estimated from multinomial logistic regression analysis. Model 1 demonstrates the simple association between women's empowerment and contraceptive use; model 2 adjusts for wealth quintiles; and the third model assesses the effect of the addition of women's socio-demographic variables.

**Table 4:** Unadjusted and adjusted odds ratios for contraceptive use by women overall empowerment and its various dimensions in household decision making.

Independent variables	Model 1 (Crude) OR (95% CI)		Model 2 (Adjusted^1^) OR (95% CI)		Model 3 (Full adjusted^2^) OR (95% CI)		Interaction significant at 0.05 level	
	Female-only	Couple	Female-only	Couple	Female-only	Couple	Women's age	Wealth Quintile
**Overall empowerment (weighted) (Score range: 0–18)**								
Independent decision	1.02 (0.99–1.06)	0.99 (0.95–1.04)	1.02 (0.98–1.05)	0.95 (0.90–1.00)	1.03 (0.99–1.07)	0.99 (0.93–1.04)	No	No
Couples' decision	1.03 (1.00–1.05)*	1.06 (1.03–1.09)***	1.02 (1.00–1.05)	1.05 (1.02–1.08)***	1.03 (1.01–1.06)*	1.06 (1.03–1.10)***	Yes	Yes
**Economic decision making (Score range: 0–6)**								
Independent decision	1.04 (0.95–1.23)	0.97 (0.87–1.08)	1.02 (0.94–1.11)	0.88 (0.78–0.99)*	1.05 (0.96–1.15)	0.94 (0.83–1.06)	No	No
Couples' decision	1.05 (0.98–1.12)	1.14 (1.06–1.22)***	1.03 (0.97–1.10)	1.11 (1.03–1.19)**	1.05 (0.98–1.12)	1.12 (1.04–1.21)**	No	Yes
**Household decision making (Score range: 0–4)**								
Independent decision	1.05 (0.92–1.19)	1.02 (0.87–1.19)	1.02 (0.89–1.16)	0.88 (0.74–1.04)	1.07 (0.93–1.23)	0.97 (0.81–1.17)	No	No
Couples' decision	1.08 (0.99–1.18)	1.30 (1.17–1.43)***	1.06 (0.97–1.16)	1.26 (1.14–1.40)***	1.10 (1.00–1.21)*	1.29 (1.15–1.44)***	Yes	Yes
**Physical mobility (Score range: 0–3)**								
Independent decision	1.21 (0.98–1.49)	0.91 (0.69–1.22)	1.19 (0.96–1.47)	0.87 (0.65–1.16)	1.26 (1.00–1.57)	1.07 (0.78–1.46)	Yes	Yes
Couples' decision	1.19 (1.04–1.36)*	1.25 (1.07–1.47)**	1.16 (1.01–1.33)*	1.21 (1.03–1.43)*	1.22 (1.06–1.41)**	1.31 (1.10–1.57)**	No	No

*p<0.05.

**p<0.01.

***p<0.001.

1 Adjusted for wealth quintile;

2 Adjusted for wealth quintile, district, women's age, age of women at marriage, husband's age, age difference, women's literacy, husband's literacy, and number of alive children.

Notes: Female-only methods include: pill, injectable, intra-uterine device, implant and female sterilization; couple methods include: condom, withdrawal, periodic abstinence and male sterilization.

The overall score for women's empowerment for independent decision-making showed no association with either female-only or couple methods in any of the three models. By notable contrast, empowerment measures for couples' joint decision-making substantially affected contraceptive use: a one-point increase in the score was related to a 1.03 times increase in the odds of using female-only methods rather than no method, and a 1.06 increase in the odds of using a couple method instead of none at all (table 4, model 1). When adjusted with wealth quintiles, the relationship held significance for only couple methods (odds ratio, 1.05). However, with the addition of women's socio-demographic variables, the measure showed significant association with both of the empowerment measures – independent and couples' decisions (odds ratio 1.03 and 1.06, respectively). Furthermore, the association changes with women's age and wealth quintile, as the interaction was found to be significant for the said relationship. With regard to the measure of economic domain, an increase of one point in the score of couples' joint decision-making was associated with a 12-per-cent increase in the odds of using couple methods as against using no method (model 3); it showed no association with female-only methods. The score of couples' joint household decisions increases the likelihood of couple methods' use rather than no method by 29 per cent (model 3) with every single unit increase in the empowerment score. Interestingly, the same measure showed no substantial relationship with the use of female-only methods in models 1 and 2, unless the model was adjusted for women's socio-demographic characteristics. We found evidence of women's age and wealth quintiles playing the role of effect modifier on the aforementioned association.

With every additional joint decision couples make about their physical mobility, the odds for using female and couple methods as against using no method increase by a factor of 1.22 and 1.31 respectively (model 3). By contrast, women's independent decision-making regarding their mobility showed no significant association with the uptake of either female-only or couple methods. Overall, the measure of couples' joint decision-making increases changes in contraceptive use - primarily in favour of couple methods: consequently, we see a greater preference for, and more use of, condoms and withdrawal methods, as has also been cited by other studies in Pakistan [27], [41].

## Discussion

Despite being one of the first countries in South Asia to start a national family-planning programme, Pakistan has had limited success in achieving desired outcomes in this area [23] as only 26% of couples use any modern contraceptive method [27]. Women's empowerment is recognized as an imperative element for improved mother-and-child health. This paper is unique among its kind as it assesses the association between various dimensions of women's empowerment and current contraception use through a structured framework. We used the data on 2,133 women interviewed in peri-urban and rural areas in three districts of Punjab.

Overall use of female-only methods versus couple methods was 10∶7, as opposed to 5∶6 nationally [42]. In general, the percentage of women with sole authority for decision-making was low. The highest level of empowerment was observed in economic decision-making, while the lowest level was observed in physical mobility, which is consistent with the previous study [16].

Factors identified as having significant associations with women's empowerment were consistent with the findings of a good many other research studies. Women alone having a final say in decision-making increases with their increased age, and that of their husbands; with an increased number of living children; with a better (socio-economic status) wealth quintile; and with women's literacy [22], [25], [35], [39], [43]. The age of women also directly links with that of their husbands and the number of children. Thus, higher decision-making power with increased age may be attributed to the cultural norm whereby a newly-married woman is expected to perform household duties under the supervision of her husband, or even mother-in-law, who is the primary decision-maker [44]. On the other hand, couples' joint decision-making correlated substantially with women's and their husbands' literacy, the number of living children, and with socio-economic status.

We observed no association between any measures of women's independent decision-making and the use of either female-only or couple contraceptive methods. By contrast, when the element of couples' joint decision-making was considered, the empowerment measures showed a substantial effect on contraceptive use. This somehow indicates the role and involvement of husbands in deciding about the use of contraception. In the main, our findings concur with evidence derived from an earlier national study in Pakistan [22], and from studies conducted in other countries [17], [20], [21]. While our findings contradict the results of another research project conducted in an urban squatter settlement in Karachi, Pakistan [23], it is pertinent to note here that the measures of empowerment and outcome classification in these studies are not the same. Unlike in previous studies, our empowerment measures were regressed on a continuous scale, constructed based on several indicators, and also classified into women's independent and couples' joint decision-making.

Within the economic domain, only the measure of couples' joint decisions substantially affects the use of couple contraceptive methods. These results are consistent with another study conducted in Pakistan [22]. Also, the association was confounded by women's status, while the wealth quintile was the effect modifier. We suggest further research investigations in order to understand the cultural phenomenon of women with unmet contraceptive needs neither opt for nor practise female-only methods, even when they have economic empowerment.

As with other measures of independent decision-making, the measure of women's independent mobility showed no association with any form of contraceptive method. However, we observed significant mediating effects of women's age and economic status on the association. This finding contradicts with the earlier qualitative study conducted in Pakistan where restricted women's mobility was identified as one of the barriers to contraceptive use [45]. A possible reason for this could be women's age: older women tend to make more independent decisions (table 3), while the chances of becoming pregnant decline with proximity to the menopause [46], which eventually lessens the need for contraception. These results may be attributed to cultural aspects whereby women in underprivileged communities receive greater encouragement to do *pardah (veil)*, and young women are usually accompanied by men and elder members of their family [16]. By contrast, women deciding about their mobility jointly with their husbands have higher chances of using female-only as well as couple contraceptive methods. The fact that this finding contradicts an earlier study carried out in Pakistan [22] could be attributed to the indicators used for the composition of this dimension. However, we suggest the area merits further research: dedicated, in particular, to studying the effect of women's independent decision-making on adopting female-only (i.e. more effective) methods, and the extent to which some women may also conceal their use of contraception from their husbands, as they require neither his support nor his involvement [47].

It is interesting to note the modest effect of overall empowerment measures as compared to the effect of each empowerment domain. In our opinion, this is due to the differences in scale (range) of each empowerment domain (see construction of women's empowerment indices above). For instance, the magnitude is highest for the measure of physical mobility which is ranged from 0–3, followed by household decision-making (score range 0–4), economic measure (score range 0–6), and, finally, overall empowerment (score range 0–18). Principally, the odds ratios for continuous variables are the ratios between individuals who are identical on the other variables but differ by one unit on the variable of interest. Therefore, in our case, the overall empowerment measures have higher range which brings more variability, thus resulting in a lower coefficient.

The key limitations of our findings are mostly related to generalizability. The data were collected through a cross-sectional survey, and only in three districts of Punjab province in Pakistan; thus the data do not yield any temporal relationship between women's empowerment and contraceptive use. Moreover, the restriction of our sample to women who were either using any form of contraception or living with unmet needs for contraception will further limit the generalizability. Secondly, married relationships are ever-changing and multi-faceted [35], details that were not quantified in our paper. Furthermore, the interviews were conducted only among women; therefore the study did not capture in-depth information about men's perspectives on fertility, or about their desire and intentions with respect to contraceptive use. Lastly, we included female sterilization in female-control methods; however, Pakistan's health policy necessitates a husband's written consent before the procedure is performed, due to the irreversible nature of it.

Despite these limitations, our study has important implications. On the whole, our findings reaffirm the fact that women with greater empowerment are more likely to use contraception. The effect of empowerment measures for couples' decision-making (overall, economic, and household empowerment) and independent decision-making (physical mobility) on contraceptive use was mediated by the women's age and wealth quintile. Importantly, couples' collective choice is more inclined towards couple contraceptive methods (such as withdrawal and condom), which are less effective. This may be the reason for the higher use of condoms and withdrawal in Pakistan – resulting in stark imbalances in the contraceptive-method mix [27]. Thus, an uplifting of the general social and cultural status of women in conservative societies like Pakistan will have a positive effect on contraceptive use. Strategies should be devised to promote the empowerment of women in relation to household, economic and physical-mobility affairs. Adopting contraceptives can help women achieve their desired goals in relation to birth spacing or limiting, in addition to ensuring that they have proper information about, and a range of, contraceptive options. We encourage family-planning programmes to engage men within the scope of their interventions, as contraceptive use rests more on couples' decisions than on women-only ones. Moreover, efforts need to be made to educate both partners equally about contraceptive methods that have higher effectiveness. In-depth research, supplemented by qualitative research, is needed to improve our understanding of decision-making within households.

## Supporting Information

Table S1 **Comparison of key family planning indicators.**
(DOCX)
