# Supplementary material for: Women's Empowerment and Contraceptive Use: The Role of Independent versus Couples' Decision-Making, from a Lower Middle Income Country Perspective
Source: PLoS One. 2014 Aug 13;9(8):e104633. doi: 10.1371/journal.pone.0104633 (PMC4131908; doi:10.1371/journal.pone.0104633)
Supplement: Table S1 — Comparison of key family planning indicators. (DOCX) [file pone.0104633.s001.docx]

| **Table S1: Comparison of key family planning indicators** | | |
| --- | --- | --- |
| **Variables** | **Original Sample**  **N=3991** | **Sample after removing women who wanted to conceive**  **N=2133** |
|  | **n (%)** | **n (%)** |
| **Contraceptive Prevalence Rate (combined)** | **762 (17.2)** | **676 (31.7)** |
| **Modern use** | **725 (16.3)** | **642 (30.1)** |
| Pill | 90 (2.0) | 81 (3.8) |
| Injectable | 132 (3.0) | 116 (5.4) |
| Condom | 248 (5.6) | 221 (10.4) |
| IUCD | 100 (2.2) | 89 (4.2) |
| Female Sterilization | 155 (3.5) | 135 (6.3) |
| **Traditional method** | **38 (0.9)** | **35 (1.7)** |
| Withdrawal | 21 (0.5) | 18 (0.9) |
| Periodic abstinence | 17 (0.4) | 17 (0.8) |
| **Unmet need for contraception** | | |
| Unmet need for contraception | 1647 (37.2) | 1451 (68.0) |
| Women with no unmet need for contraception, but using any form of contraceptive method | 210 (4.8) | 187 (8.7) |
